# Climate change and natural disasters – integrating science and practice to protect health

**DOI:** 10.3402/gha.v5i0.19295

**Published:** 2012-12-17

**Authors:** Rainer Sauerborn, Kristie Ebi

**Affiliations:** 1 Institute of Public Health, Heidelberg University, Heidelberg, Germany; 2 Umeå Centre for Global Health Research, Umeå University, Umeå, Sweden; 3 Stanford University, CA, USA

**Keywords:** climate change, natural disasters, health, health systems, development, risk management

## Abstract

**Background:**

Hydro-meteorological disasters are the focus of this paper. The authors examine, to which extent climate change increases their frequency and intensity.

**Methods:**

Review of IPCC-projections of climate-change related extreme weather events and related literature on health effects.

**Results:**

Projections show that climate change is likely to increase the frequency, intensity, duration, and spatial distribution of a range of extreme weather events over coming decades.

**Conclusions:**

There is a need for strengthened collaboration between climate scientists, the health researchers and policy-makers as well as the disaster community to jointly develop adaptation strategies to protect human.

The Intergovernmental Panel on Climate Change (IPCC) ‘Special Report on Managing the Risks of Extreme Events and Disaster to Advance Climate Change Adaptation (SREX)’ (1) was approved by the world's governments in November 2011. It constitutes a landmark assessment by a large number of scientists from a wide range of disciplines. Our article takes this report as a starting point to answer the three questions below. By doing so, we wish to add the climate change dimension to the special volume of this journal on ‘Health and Health Systems Impact of Natural Disasters’.

1. Have some types of extreme events become more frequent, severe, and longer in the past decades?
2. What is the relative contribution of climate change to the various types of extreme events?
3. How can the health community coordinate and cooperate with the climate and development communities to protect health during and after natural disasters?

Before we can answer these questions, we need to clarify the term ‘natural disaster’ and lay out our conceptual framework.

First, we would like to challenge the notion that ‘natural causes’ lead to ‘natural disasters’. In reality, there is no such thing as a purely natural disaster. The research and the policy communities agree that hazard, exposure, vulnerability, disaster risk, and adaptation are fundamentally socially constructed (2–4). All disasters include components of social choices, social constraints, and societal actions or inactions.

Second, even what we call natural causes are to some extent modified by human activities, such as many extreme hydro-meteorological and climatological events. The burning of fossil fuels and deforestation influence the frequency, intensity, duration, and spatial extent of some extreme weather and climate events, as detailed in the recent SREX report (1) and reviewed here. It is only the geophysical disasters whose causes have not been influenced by human action, yet even their impact on health and property is.

Figure 1 shows the classification of natural disasters (5) and highlights the extreme events that in principle and by plausible pathways might be influenced by climate change.

**Fig. 1:**
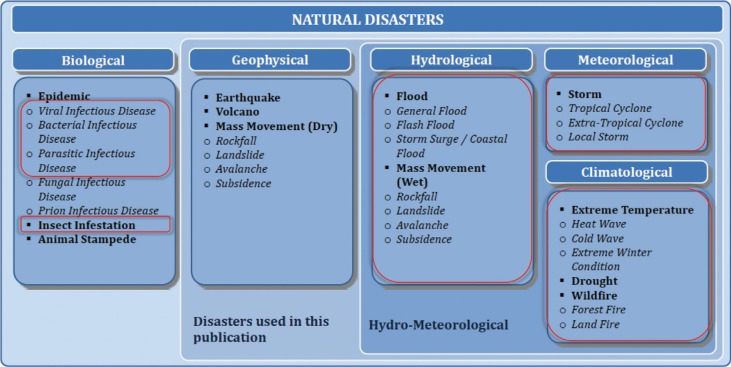
Classification of natural disasters (5). Marked with red rectangles are those groups of disasters, which can be influenced by climate change.

Hydro-meteorological and climatological events (shown in Fig. 1 in the two columns to the right) constitute the largest share of the impact of disasters on health and well-being (5). From 1996 to 2005, weather-related natural hazards caused 90% of natural disasters, with the associated deaths responsible for most of the total impacts in low-income countries (6, 7).

This article examines the contribution of climate change to hydro-meteorological and climatological disasters and the health impacts of these events. This provides opportunities for identifying interventions to reduce current vulnerability and enhance future resilience in a changing climate on both shorter and longer time scales.

## Conceptual framework

The conventional concept (1, 8, 9) frames health risks from disasters in its first approximation as a function of (i) the hazard, such as flooding or heat waves, (ii) the exposure, that is, who is exposed for how long to the hazard (e.g. to heat wave in the absence of air conditioning), and (iii) their associated vulnerability, that is, the propensity of an individual or community to be adversely affected (e.g. living in flood plains). The frequency, intensity, duration, and spatial extent of extreme weather and climate events can be reduced through mitigation of greenhouse gas emissions over the longer term, while exposure and vulnerability can be reduced in the short and longer term by adaptation strategies, policies, and measures. While exposure and vulnerability can be conceptually separated, they are closely intertwined in reality.

**Fig. 2:**
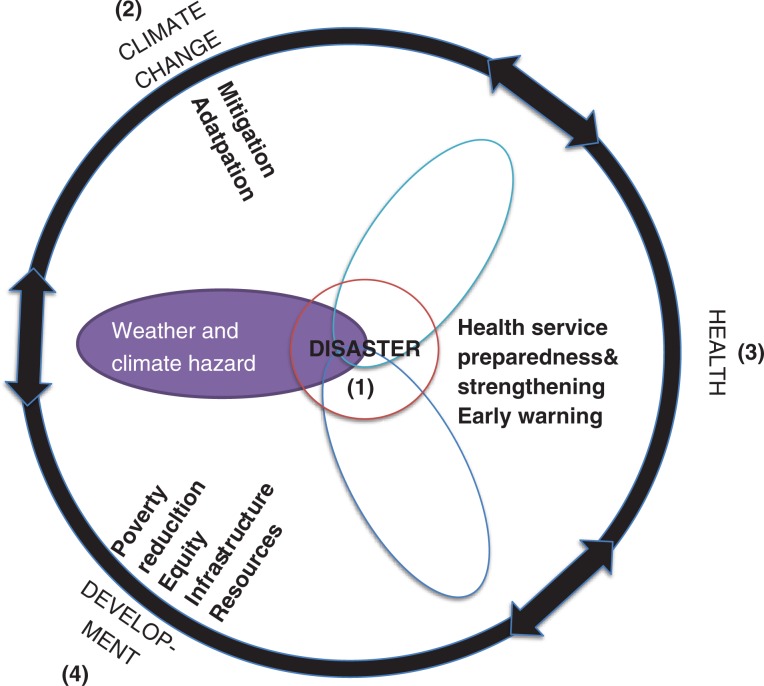
Conceptual framework of the link between natural and man-made climatic influences to disaster-related health risks. Based on SREX 2012 report, but modified and expanded.

Figure 2 provides the conceptual framework for our paper. It builds on, but modifies the concept of the SREX report (1) to include health. It illustrates the different communities involved in identifying, managing, and recovering from the health risks of extreme events: (1), climate science and climate change adaptation (2), health (3) and development (4). These sectors have multiple inter-linkages, conveyed in the black circle joining them. Hazard, exposure, and vulnerability are portrayed as petals of the flower in the center of which is disaster risk, to highlight all must be present for a disaster to occur. Inside the circle are the main actions that each of the communities for the prevention or management of health impacts of disasters.

The impacts of similar strength events in different regions have very different consequences (1). With high vulnerability, even minor events can result in extreme impacts. A strong weather or climate event can affect locations with low vulnerability. A wide range of factors, including anthropogenic climate change, natural climate variability, socioeconomic development, characteristics of the affected population, etc., influence climate extremes, exposure, and vulnerability.

In this article, we focus on adaptation strategies, policies, and measures, while noting that mitigation also is important to attenuate the contribution of climate change to hydro-meteorological disasters (e.g. 10).

We now turn to explore the evidence for answering the three questions posed earlier.

## Have some types of extreme events become more frequent, severe, and longer in the past decades?

By definition, extreme events are rare, which means the quality and quantity of data can be limited for some events. Nevertheless, observations since 1950 confirm that the frequency, intensity, spatial extent, and duration of some extreme weather and climate events have changed (Table 1) and are projected to continue to change with climate change (1). The confidence terms used in Table 1 were based on three scales: evidence and agreement; confidence; and likelihood (10). The summary terms used to describe the available evidence were limited, medium, or robust, and the degree of agreement was described as low, medium, or high. Levels of confidence were very low, low, medium, high, or very high. Likelihood was described as virtually certain (99–100%), very likely (90–100%), likely (66–100%), about as likely as not (33–66%), unlikely (0–33%), very unlikely (0–10%), and exceptionally unlikely (0–1%). The level of confidence in an observed trend neither implies nor excludes the possibility of changes in this extreme on regional scales.

**Table 1:** Observed changes in extreme weather and climate events since 1950

Event	Global change	Confidence	Regional changes	Confidence
Warm days and nights	Increase	Very likely	Increase at the continental scale in North America, Europe, and Australia	Likely
			Warming trend in daily temperature extremes in Asia	Medium
			Warming trend in daily temperature extremes in Africa and South America	Low to medium, depending on the region
Cold days and nights	Decrease	Very likely	Decrease at the continental scale in North America, Europe, and Australia	Likely
Length or number of heat waves			Increase in many regions with sufficient data	Medium
Heavy precipitation events			More regions have experienced increases than decreases, although there are strong regional and sub-regional variations	Likely
Tropical cyclone activity (intensity, frequency, duration)				Low confidence after accounting for past changes in observing capabilities
Drought			Intense and longer droughts in southern Europe and west Africa	Medium
			Less frequent, less intense, or shorter in central North America and northwestern Australia	
Extreme coastal high water	Increase related to an increase in mean sea level			Likely

There is limited to medium evidence available to assess climate-driven observed changes in the magnitude and frequency of floods at regional scales, and there is low overall confidence regarding the sign of these changes globally because the available instrumental records of floods are limited in space and time, and because of confounding effects of changes in land use and engineering.

There is limited evidence that the increase in the frequency, intensity, duration, and spatial extent of extremes have led to increases in human impacts, except for heat waves (1).

## What is the relative contribution of climate change to which type of extreme event?

Detection and attribution is a formal process for determining whether observed changes could be attributed to climate change or another factor (11, 12).

Detection is the process of determining whether climate or a system affected by climate has changed in a statistical sense, without providing a reason for that change. The question is whether there is a trend in observations that differs significantly from a previous pattern. Answering this question requires identification of a baseline from which change can be measured and a reasonably long data record. A change is detected if its likelihood of occurrence by chance is small (e.g. <10%).

Attribution is the process of evaluating the relative contributions of multiple causal factors to a change or event with an assignment of statistical confidence. Attribution is only undertaken if a change has been detected. Attribution is common in epidemiology when multiple factors each contribute to a disease burden.

Detection and attribution can be a one- or multi-step process. For climate change detection and attribution studies, four methods are commonly used (11). An approach commonly used in the climate modeling community attributes an observed change within a system to an external forcing based on explicitly modeling the response of the variable to forcing and drivers, such as greenhouse gases. Other approaches take multiple steps, for example, by first attributing an observed change in a variable of interest to a change in climate and/or environmental condition, and then separately assessing and attributing the change in climate and/or environmental condition to external drivers and forcings.

Attribution of single extreme events to anthropogenic climate change is challenging because of the rarity of the events and the inherent variability in the climate system. However, the SREX report concluded there is evidence that anthropogenic influences, including increases in atmospheric concentrations of greenhouse gases, have led to changes in some extremes at the global scale (confidence) (1):

1. warming of extreme daily minimum and maximum temperatures (likely)
2. intensification of extreme precipitation (medium)
3. increasing extreme coastal high water due to an increase in mean sea level (likely).

### Projections of extreme events

Projections of climate change-related alterations in the frequency, magnitude, duration, and spatial extent of weather and climate events for the end of the century, relative to the end of the last century, are highly uncertain. Major sources of uncertainty in projections for the end of the 21st century include the contribution of natural climate variability, projections of future greenhouse gas emissions (including projections of changes in demographics, economic growth, and technology change), uncertainties in climate model parameters and structure, and the responsiveness of the climate system to increased concentrations of greenhouse gases (1, 113). Projecting low-probability, high-impact changes is particularly challenging given understanding of climate thresholds and the coarse spatial scale of climate models. Despite these uncertainties, under all emission scenarios, projected changes are similar over the coming decades because of the inertia in the climate system, with the projected changes due to climate change relatively small compared with natural climate variability. For some extremes, it is uncertain whether the projected change in some regions might be positive or negative (e.g. increased or decreased rainfall).

Given these uncertainties, the key findings from the IPCC SREX report for the end of the 21st century include^1^:

1. Substantial increases in temperature extremes, with higher temperatures when extremes occur. At the global scale, it is virtually certain that increases in the frequency and magnitude of warm daily temperature extremes and decreases in cold extremes will occur. It is very likely that the length, frequency, and/or intensity of warm spells or heat waves will increase over most land areas. Based on the A1B and A2 emissions scenarios, a 1-in-20 year hottest day is likely to become a 1-in-2 year event by the end of the century in most regions, except in the high latitudes of the Northern Hemisphere, where it is likely to become a 1-in-5 year event. Less warming is projected under the B1 scenario, where a 1-in-20 year event would likely become a 1-in-5 year event (and a 1-in-10 year event in Northern Hemisphere high latitudes). The 1-in-20 year extreme daily maximum temperature (i.e. a value exceeded on average only once during the period 1981–2000) will likely increase by about 1°C to 3°C by the mid-21st century and by about 2°C to 5°C by the late 21st century, depending on the region and emissions scenario.
2. Increases in the frequency of heavy precipitation or the proportion of total rainfall from heavy falls, particularly in the high latitudes and tropical regions, and in winter in the northern mid-latitudes. Heavy rainfalls associated with tropical cyclones are likely to increase with continued warming. There is medium confidence that, in some regions, increases in heavy precipitation will occur despite projected decreases in total precipitation in those regions. Based on a range of emissions scenarios (B1, A1B, A2), a 1-in-20 year annual maximum daily precipitation amount is likely to become a 1-in-5 to 1-in-15 year event by the end of the 21st century in many regions, and in most regions the higher emissions scenarios (A1B and A2) project a stronger decrease in return period.
3. Increases in average tropical cyclone maximum wind speed are likely, although increases may not occur in all ocean basins. It is likely that the global frequency of tropical cyclones will either decrease or remain essentially unchanged.
4. Medium confidence in a reduction in the number of extra-tropical cyclones averaged over each hemisphere. There is low confidence in projections of small spatial-scale phenomena such as tornadoes and hail because competing physical processes may affect future trends and because of limitations in climate models to simulate them.
5. Medium confidence that droughts will intensify in some seasons and areas, due to reduced precipitation and/or increased evapotranspiration, particularly southern Europe and the Mediterranean region, central Europe, central North America, Central America and Mexico, northeast Brazil, and southern Africa. There is low confidence elsewhere. Definitional issues, lack of observational data, and the inability of models to include all the factors that influence droughts preclude stronger confidence.
6. Possible changes in floods because of projections of temperature and precipitation changes. There is low confidence in projections of changes in fluvial floods due to limited evidence and because the causes of regional changes are complex. There is medium confidence (based on physical reasoning) that projected increases in heavy rainfall would contribute to increases in local flooding in some catchments or regions.

Projections focus on how the magnitude and extent of particular extremes could change with additional climate change. Another area of concern is simultaneous events, such as occurred in South Australia in January 2009 (14–16). The region experienced a multi-year drought; in central Victoria, the 12-year rainfall totals were approximately 10–20% below the 1961–1990 average. An unprecedented heat wave occurred in January, with some of the highest temperatures ever recorded. In Victoria, there was a 25% increase in total emergency cases, with a 46% increase over the three hottest days of the heat wave. There were 980 deaths during the 4 days of the heat wave compared with a mean of 606 over the same days in the previous 5 years. A few days after the heat wave peaked, temperatures spiked again and the Forest Fire Danger Index reached unprecedented levels. High wind speeds resulted in a power line breaking, sparking a wildfire that would become one of the largest, deadliest, and most intense firestorms ever experienced in Australia's history; 173 people died. The bushfires also destroyed almost 430,000 ha of forests, crops, and pasture, and 61 businesses, causing at least AUS$4.4 billion in damage (17).

While it is impossible to project where and when increases in the frequency and intensity of a range of extreme events could occur, it is clear that when they occur (individually or jointly), these events can be challenging to effectively manage.

## How can the disaster, climate, and development communities work better with the health system community to improve disaster prevention and management?

In light of the above, there is some confidence that anthropogenic climate change will increase some hydro-meteorological disasters and their associated health impacts. It is therefore important for the disaster risk management, climate change adaptation, and climate science communities to work closely to gather evidence and develop strategies to protect health from extreme weather and climate events. One particular challenge continues to be the limited data on the health impacts of extreme weather events, from cases of infectious diseases to mental health impacts from an event itself or from the recovery process.

Although the disaster risk management and climate change adaptation communities have similar goals of increasing resilience to extreme events, a challenge has been that the two communities have worked in parallel, using different frameworks and definitions, with different institutional set-ups, funding sources, conferences, and assessments. The disaster risk management community has extensive experience with designing, implementing, and evaluating strategies, policies, and measures to improve understanding of disaster risk, enhance disaster risk reduction and transfer, and continuously promote improvement in disaster preparedness, response, and recovery. Until recently, disaster risk management did not consider how climate change could alter the effectiveness of their programs. Of necessity, their focus is on preventing, responding to, and recovering from a range of events, not all climate-related, with less attention to longer-term risks. The adaptation community has experience with incorporating consideration of current and projected climate change into policies and programs to increase resilience of communities and regions, to moderate harm and exploit beneficial opportunities. Bringing these communities together provides opportunities to learn from each other, identifying lessons learned and best practices.

The development community also should be engaged in efforts to better manage the risks of extreme events and disasters because development or lack thereof is a key modifier of exposure and vulnerability. Table 2 estimates the effect that various disasters might have on the achievement of the millennium development goals (MDGs) (18). Underdeveloped communities and countries will be much more vulnerable to climate change impacts, particularly in health.

**Table 2:** Hydro-meteorological disasters by type of health impact and their impact on the attainment of the millennium development goals (MDGs)

	Stroms	Floods	Heart	Drought	MDG
No. of deaths	Few	Many	Moderate	Many	5
No. of severe injuries	Few	Few	n.a.	n.a.	
Pregnancy/delivery	Low	Moderate	Moderate	Likely	4
Education	Low	Moderate	Low	Moderate	3
NCDs	Widespread	Widespread	Widespread	Widespread	n.a.
Pests and vectors	Widespread	Widespread	Unlikely	Low	7
Food scarcity	Low	Moderate	Low	Likely	2
Risk of epidemics	Unlikely	Moderate	Unlikely	Unlikely	
Clean water	Widespread	Widespread	Unlikely	Widespread	7
Sanitation	Widespread	Widespread	Unlikely	Unlikely	7
Health infrastructure	Widespread	Widespread	Unlikely	Unlikely	4, 5, 7
Migration	Unlikely	Unlikely	Unlikely	Likely	
Loss of shelter	Moderate	Likely	Unlikely	Unlikely	

Frequent calls have been made to bring together the disaster risk management, climate change adaptation, climate science, and development communities to jointly assess impacts and design adaptation strategies (19–22); however, few studies focus on the health sector as a fourth partner (see Fig. 2). Exceptions include (23, 24). Ebi (25) describes an all-hazards approach in the United States that could be modified to include the additional risks of climate change. The SREX report includes specific suggestions for improved disaster risk reduction and climate change adaptation in the health sector (1). A range of options exist to increase resilience to the health risks of extreme weather and climate events, from improving health surveillance to implementing early warning systems. Better tracking of health data during and after extreme events is critically important to improve the evidence base on morbidity and mortality, which will help improve the design of early warning systems by identifying populations and regions at increased risk. Although there is general understanding about which subgroups are more vulnerable to specific events, the lack of specific information means that early warning systems are missing opportunities to ensure that those most at risk know appropriate actions to reduce the likelihood of suffering adverse impacts.

Extreme weather and climate events continue to cause preventable injuries, illnesses, and deaths. Projections that climate change is likely to increase the frequency, intensity, duration, and spatial extent of some extreme events over coming decades is added incentive to strengthening ties between the health sector and the research and practice communities focused on adaptation, disaster risk management, development, and climate science.

Such intersectoral cooperation should have at least two key components:

1. the ability to quickly implement uniform health surveillance responses in the aftermath of major ‘natural’ disasters
2. data collection to support evaluations of the extent to which disaster-related health impacts could be attributed to climate change.

Coordination and collaboration within an all-hazard approach to managing the health risks of extreme events can prevent further adverse impacts and increase resilience to other risks.

## Footnotes

1 Text is nearly identical to that in the SREX report; it was slightly edited for clarity and brevity.
